# Functional Investigation of the Receptor to the Major Pheromone Component in the C-Strain and the R-Strain of the Fall Armyworm *Spodoptera frugiperda*

**DOI:** 10.3390/insects16030304

**Published:** 2025-03-14

**Authors:** Arthur Comte, Alizée Delarue, Marie-Christine François, Christelle Monsempes, Camille Meslin, Nicolas Montagné, Emmanuelle Jacquin-Joly

**Affiliations:** 1 Institute of Ecology and Environmental Sciences of Paris (iEES-Paris), INRAE, Sorbonne Université, CNRS, IRD, UPEC, Université Paris Cité, 78026 Versaille, France; arthur.comte@inrae.fr (A.C.); alizee.delarue@universite-paris-saclay.fr (A.D.); marie-christine.francois@inrae.fr (M.-C.F.); christelle.monsempes@inrae.fr (C.M.); camille.meslin@inrae.fr (C.M.); 2 Institut Universitaire de France, France

**Keywords:** sex pheromone receptor, host-strains, single-sensillum recordings, modeling, fall armyworm, *Spodoptera frugiperda*

## Abstract

The fall armyworm *Spodoptera frugiperda* (Lepidoptera: Noctuidae) is an important invasive polyphagous crop pest that has been invading the world since 2016. This species consists of two strains adapted to different host plants, the corn strain and the rice strain, which also exhibit differences in their mating behavior, pheromone composition, and pheromone receptor sequences. A way to monitor invasion and control this pest is the use of synthetic sex pheromones to trap adults and disturb the mate-finding process via the release of large amounts of pheromones in the air. However, the efficiency of these methods depends on the specificity and sensitivity of the corresponding pheromone receptors. Yet, only pheromone receptors of the corn strain have been characterized, and nothing is known about the specificity and sensitivity of the rice strain orthologues. To address this gap, we functionally expressed the receptors to the major sex pheromone component of the two strains in *Drosophila* olfactory sensory neurons and challenged them with a large panel of pheromone compounds using single-sensillum recordings. Although their sequences present subtle mutations, we revealed that they share similar response spectra and sensitivity. The implications of these results on pheromone-based pest management strategies are discussed.

## Simple Summary

The fall armyworm, *Spodoptera frugiperda*, is a major insect pest worldwide whose monitoring is essential to detect populations and prevent new invasions. An efficient way of population monitoring is the use of sex pheromone lures. However, variation in the responses of *S. frugiperda* males to female sex pheromone compounds has been observed that may affect the monitoring process. Here, we identified mutations in the protein sequence of the odorant receptor tuned to the major sex pheromone component of *S. frugiperda* between the known Corn and Rice strains and investigated if they could explain the variation in the insect response. We did not reveal any difference in selectivity or sensitivity of the odorant receptors, suggesting that other pheromone receptors tuned to minor components of the pheromone blend are involved. A better knowledge of the functional properties of *S. frugiperda* pheromone receptors may help optimize synthetic pheromone lures, a critical step in the efficient monitoring and control of this global pest.

## 1. Introduction

Sex pheromones are used by many moth species for mating purposes (see https://pherobase.com/, accessed on 2 January 2025). These pheromones usually consist of a blend of several volatile compounds emitted by the females to attract conspecific males at a distance for mating [1]. The blend composition and component ratio are species-specific, ensuring species isolation [2]. The pheromone components are detected by olfactory sensory neurons (OSNs) located in the dedicated sensory sensilla, the pheromone-sensitive sensilla, carried by male antennae [3]. These OSNs detect the pheromone components via a specific class of odorant receptors (ORs), the pheromone receptors (PRs) [4]. Like ORs, PRs are seven transmembrane domain proteins expressed at the dendritic membrane of the OSNs. They function via complex formation with an obligatory co-receptor conserved across insect species and named Orco [5]. Together, PR and Orco form an ion channel that opens upon pheromone binding, leading to membrane depolarization [6,7]. The pheromone signal is thus transformed into an electrical signal that is transmitted to the brain for signal integration and interpretation, further leading to the attraction behavior. Whereas the majority of ORs have a broad activation spectrum, i.e., they can recognize several odorant molecules, PRs are often very specific to one component of the pheromone blend or at least narrowly tuned to similar chemicals [8].

To date, numerous moth PRs have been functionally characterized (meaning the pheromone compound that activates them is identified) [4], including that of the fall armyworm *Spodoptera frugiperda* [9,10,11,12]. This polyphagous Noctuid species is one of the major crop pests in the world: originating from the Americas, in recent years, it invaded Africa (2016), then India (2018), China (2019), Australia (2020), and it is now found in the Mediterranean basin, including some European countries [13]. The voracious larvae cause damage to a large variety of food crops [14], causing drastic economic losses. In its native range, this species presents genetically distinct strains exhibiting several prezygotic isolation barriers [15], the corn (C) and rice (R) strains. One feeds preferentially on large grasses like corn, cotton, and sorghum (C-strain) while the second is usually associated with rice and other small grasses (R-strain) [16]. The two strains are indistinguishable morphologically, but they differ in their dispersal patterns, population structure, and genomic signatures of selection on regions potentially involved in reproductive isolation and insecticide resistance [15,17]. In particular, they exhibit differences in their mating behavior and pheromone compositions. The females of both strains produce (*Z*)-9-tetradecenyl acetate (abbreviated as (*Z*)-9-14:OAc, the major component in both strains), (*Z*)-11-16:OAc, (*Z*)-9-12:OAc and (*Z*)-7-12:OAc in their pheromone gland that are attractive to males in field experiments [18] and the difference between strains relies on relative amounts of some of these components [19]. On the receiver side, several factors may correlate with variations in pheromone composition to ensure correct male preference, such as differences in the sequence of the corresponding ORs, in their expression levels, in OSN numbers, in the functional topology of the antennal lobe, or in the development of neuronal pathways [20,21], but this has not been yet investigated in the C-and R-strains of *S. frugiperda*. In this work, we focused on the contribution of pheromone receptor sequence variation. Among the *S. frugiperda* ORs (SfruORs) functionally characterized and tuned to pheromone compounds, SfruOR13 is the receptor to (*Z*)-9-14:OAc, with almost equal response to the heterologous pheromone compound (*Z,E*)-9,12-14:OAc, and minor response to (*Z*)-9-12:OAc [9,10], and SfruOR56 and SfruOR62 are both tuned to (*Z*)-7-12:OAc [10]. These studies focused on SfruORs from the C-strain, and the orthologues from the R-strain have not been investigated yet.

A way to monitor *S. frugiperda* invasion and control populations is the use of synthetic sex pheromones [15]. Pheromone lures are used to trap *S. frugiperda* adults to determine the best timing of pesticide applications [22], and they can also be used for mass trapping to reduce populations [23]. Mating disruption consists of releasing large amounts of pheromone in the air to disturb the male capacities to detect females [24]. The efficacy of these different strategies depends on the ability of the males to respond to the synthetic pheromone. In *S. frugiperda*, it has been shown that the efficacy of predefined sex pheromone traps varies across different geographic regions [25] and that sex pheromone composition differs among populations from various areas [26]. This variation raises questions about the uniformity of response to a single synthetic pheromone blend across different strains or populations. Notably, subtle amino acid substitutions in pheromone receptor sequences can lead to significant changes in their functionality [27,28], potentially impacting trapping strategies, compromising accurate monitoring, and reducing the efficiency of mating disruption efforts. Therefore, we hypothesize that the specificity and sensitivity of pheromone receptors may differ between host strains.

To investigate this, we aim to determine whether both C- and R-strains utilize identical PRs with the same properties for detecting sex pheromones. We took advantage of the published genomes of both strains [29] to compare the amino acid sequences of the four SfruORs involved in sex pheromone detection. We focused on SfruOR13, which presented the highest number of amino acid mutations (six) between orthologous OR pairs, and is crucial in sex pheromone detection, as it detects the major pheromone component of the blend. We compared the response spectra and sensitivity of both C- and R-SfruOR13 and revealed no difference in qualitative response spectra or sensitivity to the major pheromone component (*Z*)-9-14:OAc and other pheromone ligands.

## 2. Materials and Methods

### 2.1. Comparative Sequence Alignment and Structural Mutation Mapping of C- and R-Strain SfruOR13

The SfruOR13 sequences (GSSPFG00011660001.3-PA and SFRICE002163.1-PA for the C-strain sequence and the R-strain sequence, respectively) were retrieved from the *S. frugiperda* genome v3.1 of the C-variant (annotation OGS2.0) and v1.0 of the R-variant (annotation OGS2.2) available on the BIPAA website (https://bipaa.genouest.org/is/, accessed on 2 January 2025) [29]. Sequences were aligned using MAFFT web-based version 7.220 [30] with default parameters. The receptor orientation in the membrane was determined using PPM 3.0 server [31]. Protein models for both C- and R-strains of SfruOR13 were generated using AlphaFold2 [32], selecting the most reliable relaxed model based on the highest pLDDT score from five generated models. The pLDDT scores of the residues in the selected models were assessed to ensure that the vast majority exhibited a high confidence level (pLDDT > 90), with particular focus on those within the predicted binding region (Figure S1A). To further validate the reliability of our models, we conducted structural alignments using PyMOL 2.5.4 [33], comparing the C-strain and R-strain SfruOR13 models with published Cryo-EM structures of apo insect ORs (Protein Data Bank accession codes: 7LIC, 8V00, 8V3C, and 8Z9Z) [6,7,34] (Figure S1B). To determine the binding region of SfruOR13s, their models were superimposed with Cryo-EM structures of *Machilis hrabei* (Mhra) OR5 in complex with eugenol and DEET (Protein Data Bank accession 7LID and 7LIG) [34]. After visual inspection of the cavities identified by Fpocket 4.0 [35], the main cavity coinciding with the MhraOR5 binding site was proposed as the putative binding site in each SfruOR13 model. Residues within 5 Å from the surface of the main cavity were considered part of the binding region.

### 2.2. Heterologous Expression of C- and R-Strain SfruOR13 in Drosophila melanogaster

The SfruOR13 full-length ORFs were synthesized in vitro and sub-cloned into the *pUAST.attB* vector by Synbio Technologies (Monmouth Junction, NJ, USA). EndoFree *pUAST.attB*-*SfruOR13* plasmids were injected into fly embryos of the genotype y^1^ M{vas-int.Dm}ZH-2A w*; M{3xP3-RFP.attP’}ZH-51C to generate *D. melanogaster UAS*-*SfruOR13* lines (BestGene Inc., Chino Hills, CA, USA). Transgenic *Drosophila* expressing *SfruOR13* transgenes in the at1 trichoid sensilla (genotype *w*; *UAS-SfruOR13*; *Or67d^GAL4^*) were obtained by crossing flies from the *UAS-SfruOR13* lines with flies from the *Or67d^GAL4^* mutant knock-in line [36]. Flies were reared on a standard medium composed of cornmeal, yeast, and agar at 25 °C, under a 12 h:12 h light–dark cycle.

### 2.3. Single-Sensillum Recordings and Pheromone Compounds

Single-sensillum recordings (SSRs) were performed on at1 sensilla of 2-to 5-day-old *Drosophila* as described in de Fouchier et al., 2015 [37]. Stimulus cartridges consisted of Pasteur pipettes containing a filter paper loaded with 10 µL of *n*-hexane solution of each pheromone compound. Control cartridges contained 10 µL of hexane alone. 11-cis-vaccenyl acetate (cVA) was also used as a control to ensure the absence of OR67d in at1 sensilla, cVA being the major ligand of *Drosophila* OR67d [38]. For the screening experiment, a panel of 26 pheromone compounds, including the components of the *S. frugiperda* sex pheromone (*Z*)-9-14:OAc, (*Z*)-11-16:OAc, (*Z*)-9-12:OAc, and (*Z*)-7-12:OAc, was used to compare the response spectra of the C- and the R-SfruOR13. Ten µL of a 1 µg·µL^−1^ hexane solution of each of the 26 compounds were loaded on the filter paper. Source and purity of the pheromone compounds used as stimuli can be found in Table S1. For each of the odorants identified as ligands for one or both SfruOR13s, dose–response analyses were performed using the same protocol as described above with decimal dilutions ranging from 1 ng·µL^−1^ up to 1 µg·µL^−1^.

The activity of at1 neurons was amplified using an EX-1 amplifier (Dagan, Minneapolis, MN, USA), digitized through Axon Digidata 1550A Data Acquisition System (Molecular Devices, Sunnyvale, CA, USA), recorded and analyzed using the pCLAMP 11 software (Molecular Devices). Net responses of at1 neurons expressing SfruOR13 from C- and R-strains were determined by subtracting the spontaneous firing rate from the firing rate during the odorant stimulation, following the methodology outlined in de Fouchier et al., 2017 [14]. An odorant was considered as a ligand if the response was statistically different from the response induced by the solvent alone (Kruskal–Wallis test, followed by a Dunn’s multiple comparison test adjusted with Benjamini and Hochberg correction, *p* < 0.05). All statistical analyses were performed using R (version 4.3.2).

## 3. Results

### 3.1. The Binding Regions of SfruOR13s Are Conserved Between the C- and the R-Strains

**Figure 1:**
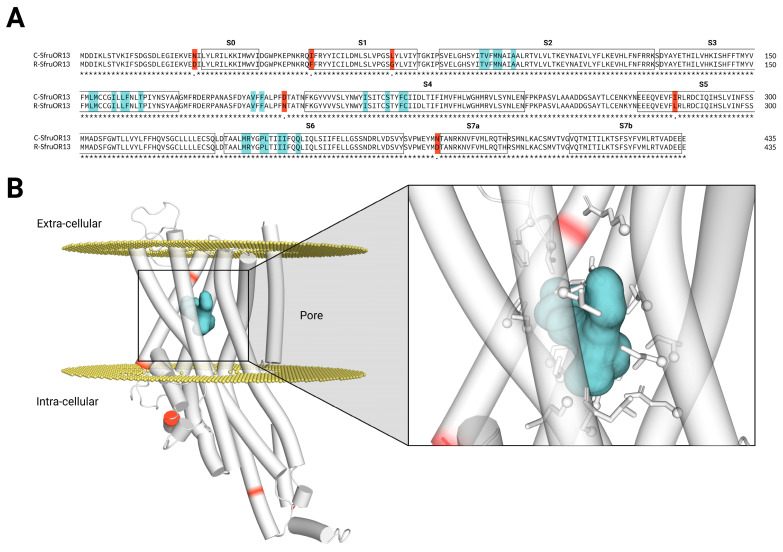
The predicted binding region of SfruOR13 is conserved between the C- and the R-strains of *S. frugiperda*: (**A**) Sequence alignment of SfruOR13s from the C- and the R-strains (identity = 98.6%). The helices (S) are indicated by black boxes. Amino acids that differ between the two strains are highlighted in red, while the residues predicted to be part of the binding region are highlighted in blue. (**B**) Three-dimensional structural representation of the C-strain SfruOR13, with residues differing between C- and R-strains highlighted in red. The predicted binding region volume is shaded in blue, and the side chains of the predicted binding region residues are displayed as stick models. The receptor orientation in the membrane (yellow) was determined using PPM 3.0 server [31]. The 3D structural representation was visualized using PyMOL 2.5.4 [33].

The proteins encoded by the *SfruOR13* genes from the *S. frugiperda* C-and R-strain genomes both contained 435 amino acids and shared 98.6% sequence identity, differing by only six amino acids (Figure 1A). To explore whether these substitutions might affect ligand detection or receptor activation, 3D models of C- and R-SfruOR13 were generated, and these mutations were mapped onto the structures. The rationality of these models was validated using the pLDDT scoring function included in AlphaFold2 and structurally aligning these models with the published Cryo-EM structures of apo insect ORs [6,7,34] (Figure S1A,B). Superimposition of the C-SfruOR13 and R-SfruOR13 models in PyMOL revealed striking structural similarities between the two receptors (RMSD = 0.142 Å). Moreover, the six differing residues between the corn and rice sequences were located far from the predicted binding region (T90, V91, M93, N94, A97, L153, M154, I158, L160, F161, T164, V189, F191, I214, S219, F222, C223, M337, R338, P341, L342, I345, I346, Q349; Figure 1A), as well as the transmembrane helical segments involved in pore formation within the tetrameric complex with Orco (Figure 1A,B). These results suggest that these substitutions are unlikely to affect receptor function. To test this hypothesis, we experimentally analyzed the response spectrum of each SfruOR13.

### 3.2. C- and R-SfruOR13 Exhibit the Same Specificity and Sensitivity to Pheromone Compounds

**Figure 2:**
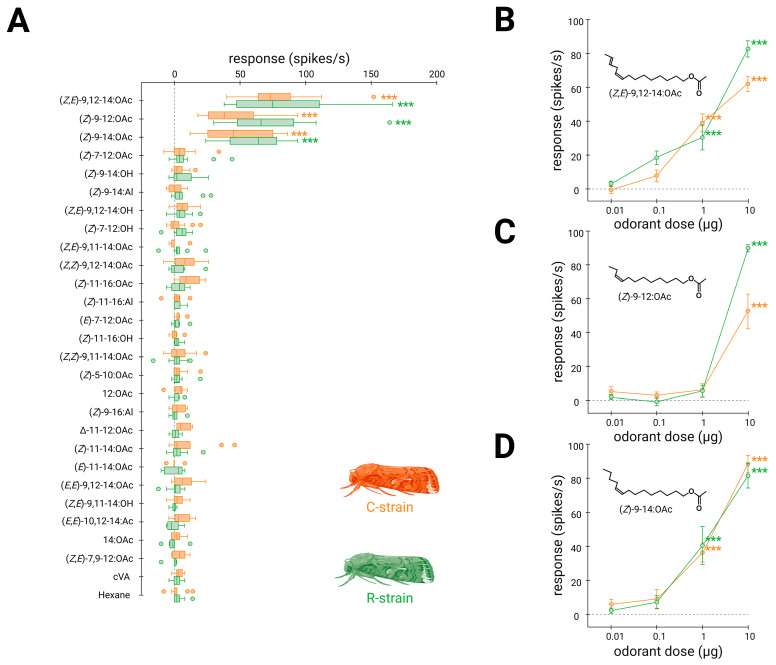
C- and R-SfruOR13 exhibit the same specificity and sensitivity to pheromone compounds: (**A**) Box plot showing the responses of *Drosophila* at1 OSNs (*n* = 5–13) expressing either C- or R-SfruOR13 measured upon exposure to a panel of pheromone compounds (vertical line; 10 µg loaded in the stimulus cartridge). cVA and solvent are used as controls. The whiskers were extended to 1.5 times the interquartile range from the quartiles. Outliers are indicated by dots. Orange boxes represent the responses of SfruOR13 from the C-strain and green boxes indicate the responses of SfruOR13 from the R-strain. (**B**–**D**) Dose–response curves of *Drosophila* at1 OSNs for all active compounds revealed during the screening of the panel. Data represented are mean action potential frequencies ± SEM (*n* = 4–9). In (**A**–**D**), asterisks indicate statistically significant differences between responses to the odorant and to the solvent (Kruskal–Wallis test, followed by a Dunn’s multiple comparison test adjusted with Benjamini and Hochberg correction; *** *p* < 0.001).

To compare the response spectra of the two SfruOR13 variants, we generated two *D. melanogaster* lines expressing each of these variants in at1 OSNs instead of the endogenous receptor DmelOR67d. Antennae of transgenic flies were stimulated with a large range of pheromone compounds, including the four components of the *S. frugiperda* sex pheromone, pheromone compounds from other *Spodoptera* species, and related compounds (Table S1). Single-sensillum recordings showed that C-SfruOR13 was significantly activated by the same three compounds that elicited a response in R-SfruOR13: (*Z*,*E*)-9,12-14:OAc, (*Z*)-9-12:OAc, and (*Z*)-9-14:OAc (Figure 2A).

The three active compounds were tested in dose–response experiments to compare the response threshold of the two SfruOR13 variants. For each selected odorant, 4 decimal dilutions ranging from 1 µg·µL^−1^ to 1 ng·µL^−1^ were used to generate the stimulation cartridges. Both C- and R-SfruOR13s showed a significant response to (*Z*)-9-12:OAc only at a dose of 10 μg, whereas they were still activated by (*Z,E*)-9,12-14: OAc and (*Z*)-9-14:OAc at 1 μg (Figure 2B–D).

These single-sensillum recordings confirmed that the two SfruOR13 variants exhibited identical specificity and selectivity for the tested panel of pheromonal compounds.

## 4. Discussion

Precise detection and recognition of the sex pheromone is crucial for successful mating and species survival. Subtle mutations in pheromone receptors can affect the receptor response profile and thus can disrupt sexual communication and potentially lead to speciation. For instance, a single residue mutation in the third transmembrane domain of orthologous PRs in two *Ostrinia* species, *O. nubilalis* and *O. furnacalis*, has been shown to alter the sensitivity of the receptor to pheromone components [27]. In *Helicoverpa* sister species, *H. armigera* and *H. assulta*, two amino acids also located in the intracellular domains determine together the functional difference between two orthologous PRs [28]. Up to eight mutations in the binding pocket of PRs can alter their response spectra from broadly tuned to narrowly tuned, as recently demonstrated in *Spodoptera* spp. by ancestor PR gene reconstruction, functional studies of encoded proteins, and site-directed mutagenesis [39]. From a pest control perspective, variation in the response of PRs to sex pheromones has the potential to affect pheromone-based monitoring and management of pest species, since the efficacy of these strategies depends on the ability of the receiver to respond to the synthetic pheromone. In the case of invasive species like the fall armyworm *S. frugiperda*, efficient and timely monitoring is particularly crucial.

In *S. frugiperda*, two genetically differentiated strains co-exist, the so-called C-strain and R-strain, and variation in male responses to synthetic sex pheromone has been reported between the two strains [19], as well as between geographic populations [25,40], with a possible impact on population monitoring. We thus wondered if such variation may result from differences in the PR sequences, possibly impacting their specificity and/or sensitivity. As the genomes of the two host-plant strains have been sequenced [29], we investigated this question at the strain level. Mining the two genomes allowed us to retrieve the C-and R-orthologous genes encoding SfruOR13, the receptor previously characterized as tuned to (*Z*)-9-14:OAc [9,10], the major component of the sex pheromone blend in the two strains [18]. Amino acid sequences of the two proteins differed by six amino acids, half of them being located in transmembrane domains. Interestingly, the two studies that previously investigated the response spectrum of SfruOR13 were conducted only on the C-strain. None of these studies specified the strain used, but the analysis of the SfruOR13 sequence provided by Guo et al. (2020) [9] revealed that the six critical amino acids correspond to those of the C-strain. In the second study [10], the sequence is not provided but we suspect that it corresponds to the C-strain. Indeed, the SfruOR13 gene has been amplified in this study from a population originating from a cornfield in Yunnan Province, China, and invasive *S. frugiperda* populations, including those in China, have been shown to originate from the C-strain [41]. Thus, the function of SfruOR13 from the R-strain has not been explored yet.

To fill this gap, we first conducted in silico analyses to predict the structure and membrane domains of the two SfruOR13 orthologues. Thanks to the recent advances in the establishment of insect OR tri-dimensional structures [6,7,34,42] coupled with the rise of AlphaFold [32], a revolutionary tool for 3D protein structure modeling, it is now possible to accurately predict insect OR structures. The predicted general topology and membrane domains of both SfruOR13s were highly similar and corresponded to those observed in previous studies on insect ORs [6,7,34,42,43]. Next, we compared their binding domains, as amino acid differences may suggest different binding abilities to pheromone compounds. The predicted binding regions of SfruOR13s from the C- and the R-strains were identical. The six amino acid mutations were all predicted to be outside the binding region, suggesting that the two orthologues share a similar odor space. Additionally, these substitutions do not seem to play a role in receptor activation, as they do not impact the transmembrane domains involved in the formation of the ion channel within the tetrameric complex [6,7,34,42].

To test this hypothesis, we heterologously expressed the two SfruOR13 orthologues in *Drosophila* OSNs from at1 sensilla, a system that has proven to be highly efficient for the functional characterization of many moth PRs [4]. When challenged with a panel of 26 pheromone compounds, including the four components of the *S. frugiperda* sex pheromone blend, we did not evidence differences in qualitative and quantitative responses of the two SfruOR13s, confirming our hypothesis. As previously reported [9,10], we found that SfruOR13 from the C-strain responded to the three compounds (*Z*,*E*)-9,12-14:OAc, (*Z*)-9-12:OAc and (*Z*)-9-14:OAc, with higher sensitivity to both (*Z*)-9-14:OAc and (*Z,E*)-9,12-14:OAc. It has to be noticed that the two previous studies used two different expression systems: one used *Xenopus* oocyte expression coupled to two-voltage clamp electrophysiology [9], while the other used the same expression system as in the current study (*Drosophila* at1 OSNs coupled to SSR) [10]. Whereas it is known that the expression system can alter the response spectrum or the sensitivity of a given insect OR [44,45], no difference was found for SfruOR13 from the C-strain in the different studies, suggesting that we can be confident in the results. In addition, an OSN type identified via SSR in *S. frugiperda* type I trichoid sensilla carried by male antennae has been shown to respond to the same three compounds with similar sensitivity [10,26]. Thus, we are confident that SfruOR13 is the OR expressed in this sensillum type.

Comparing the response spectrum of SfruOR13 from the R-strain with that of the C-strain, we did not see evidence of any difference: both orthologues were tuned to the same pheromone compounds, with equal sensitivities. Since the six amino acid mutations were all predicted to be outside the binding region of SfruOR13, the simplest hypothesis is that the six mutated amino acids are not involved in the binding of the pheromone compounds, thus not impacting the pheromone recognition process. The amino acid mutations may consist of strain polymorphism without functional impact.

Our results suggest that variation in the male responses to the sex pheromone between the two *S. frugiperda* strains is not due to the differences observed in the orthologous SfruOR13 sequences. Variations in the expression level of SfruOR13 or in the number of SfruOR13-expressing OSNs between strains may participate in these differences, which could be investigated using comparative electrophysiology between *S. frugiperda* males from the different strains. In addition, the volume of the brain regions targeted by the SfruOR13-expressing OSNs or the development of neuronal pathways may differ, as observed in the two pheromone strains of *Ostrinia nubilalis* that present different preferences for the same pheromone compounds [20,21]. Alternatively, since other sex pheromone receptors have been characterized in the C-strain, namely SfruOR6, 16, 56, and 62 [9,10], a comparison of their expression levels and functional properties with those of their orthologues from the R-strain awaits to be conducted to help interpret the observed variation in male responses. Similarly, geographic variation could be investigated, possibly pinpointing mutation in orthologous PRs from different populations. A better knowledge of the functional properties of *S. frugiperda* PRs may help optimize synthetic pheromone lure, a critical step in efficient monitoring and control of this global pest and in preventing new invasions.

## Data Availability

The SfruOR13 sequences used in this study are available as GSSPFG00011660001.3-PA and SFRICE002163.1-PA for the corn strain sequence and the rice strain sequence, respectively, in the *S. frugiperda* genome v3.1 of the corn variant (annotation OGS2.0) and v1.0 of the rice variant (annotation OGS2.2) on the BIPAA website (https://bipaa.genouest.org/is/, accessed on 2 January 2025).

## Supplementary Materials

The following supporting information can be downloaded at https://www.mdpi.com/article/10.3390/insects16030304/s1, Table S1: Source and purity of the pheromone compounds used in this study. Figure S1: The AlphaFold2 models of SfruORs suggest high structural quality, particularly in the binding region, as highlighted by their pLDDT scores: (**A**) AlphaFold2 best models of C-SfruOR13 and R-SfruOR13 colored according to pLDDT scores: blue (≥90), yellow (70–90), and orange (<70). The side chains of the residues in the predicted binding region are shown as sticks. The receptor orientation in the membrane was determined using PPM 3.0 server [31] This figure was generated using the molecular visualization software PyMol 2.5.4 [33]. (**B**) Structural alignment of the best models of C-SfruOR13 and R-SfruOR13 with the published Cryo-EM structures of apo insect ORs [6,7,34] was performed using PyMOL. RMSD values were calculated across all receptor sequences to assess structural similarities.
