# Supplementary material for: Functional Investigation of the Receptor to the Major Pheromone Component in the C-Strain and the R-Strain of the Fall Armyworm Spodoptera frugiperda
Source: Insects. 2025 Mar 14;16(3):304. doi: 10.3390/insects16030304 (PMC11943435; doi:10.3390/insects16030304)
Supplement: Supplementary file 1 [file insects-16-00304-s001.zip › insects-3481732-supplementary.pdf]

Table S1. Source and purity of the pheromone compounds used in this study.

Full Name	Abbreviation	CAS	Source	Purity
(*Z*,*E*)-9,11-tetradecadienyl acetate	(*Z*,*E*)-9,11-14:OAc	50767-79-8	Pherobank	>96.8%
(*Z*,*Z*)-9,11-tetradecadienyl acetate	(*Z*,*Z*)-9,11-14:OAc	54664-98-1	*Synthesized in the lab*	95%
(*E*)-11-tetradecenyl acetate	(*E*)-11-14:OAc	33189-72-9	Pherobank	>99%
(*Z*)-11-hexadecenyl acetate	(*Z*)-11-16:OAc	34010-21-4	*Synthesized in the lab*	95%
(*Z*)-11-tetradecenyl acetate	(*Z*)-11-14:OAc	20711-10-8	Pherobank	>99%
(*Z*)-9-dodecenyl acetate	(*Z*)-9-12:OAc	16974-11-1	*Synthesized in the lab*	95%
11-dodecenyl acetate	Δ11-12:OAc	35153-10-7	*Synthesized in the lab*	95%
(*E*,*E*)-9,12-tetradecadienyl acetate	(*E*,*E*)-9,12-14:OAc	-	Pherobank	98%
(*Z*)-7-dodecenyl acetate	(*Z*)-7-12:OAc	14959-86-5	Sigma	95%
(*Z*,*Z*)-9,12-tetradecadienyl acetate	(*Z*,*Z*)-9,12-14:OAc	51354-22-4	Pherobank	98%
tetradecyl acetate	14:OAc	638-59-5	Pherobank	>99%
(*Z*)-11-hexadecen-1-ol	(*Z*)-11-16:OH	56683-54-6	*Synthesized in the lab*	95%
(*Z*,*E*)-9,12-tetradecadienyl acetate	(*Z*,*E*)-9,12-14:OAc	30507-70-1	Pherobank	>98.4%
(*Z*)-9-tetradecenyl acetate	(*Z*)-9-14:OAc	16725-53-4	Pherobank	>99%
(*Z*,*E*)-7,9-dodecadienyl acetate	(*Z*,*E*)-7,9-12:OAc	55774-32-8	*Synthesized in the lab*	95%
dodecyl acetate	12:OAc	112-66-3	*Synthesized in the lab*	99%
(*Z*)-7-dodecen-1-ol	(*Z*)-7-12:OH	20056-92-2	*Synthesized in the lab*	95%
(*E*)-7-dodecenyl acetate	(*E*)-7-12:OAc	16677-06-8	*Synthesized in the lab*	95%
(*Z*)-5-decenyl acetate	(*Z*)-5-10:OAc	67446-07-5	Sigma	95%
(*Z*,*E*)-9,12-tetradecadien-1-ol	(*Z*,*E*)-9,12-14:OH	51937-00-9	*Synthesized in the lab*	92%
(*Z*)-9-tetradecenal	(*Z*)-9-14:Al	53939-27-8	*Synthesized in the lab*	96%
(*Z*)-11-hexadecenal	(*Z*)-11-16:Al	53939-28-9	Pherobank	97%
(*Z*)-9-hexadecenal	(*Z*)-9-16:Al	56219-04-6	Pherobank	96%
(*Z*,*E*)-9,11-tetradecadien-1-ol	(*Z*,*E*)-9,11-14:OH	63025-02-5	Pherobank	96%
(*Z*)-9-tetradecen-1-ol	(*Z*)-9-14:OH	35153-15-2	*Synthesized in the lab*	98%
(*E*,*E*)-10,12-tetradecadienyl acetate	(*E*,*E*)-10,12-14:OAc	69775-61-7	*Synthesized in the lab*	95%

A

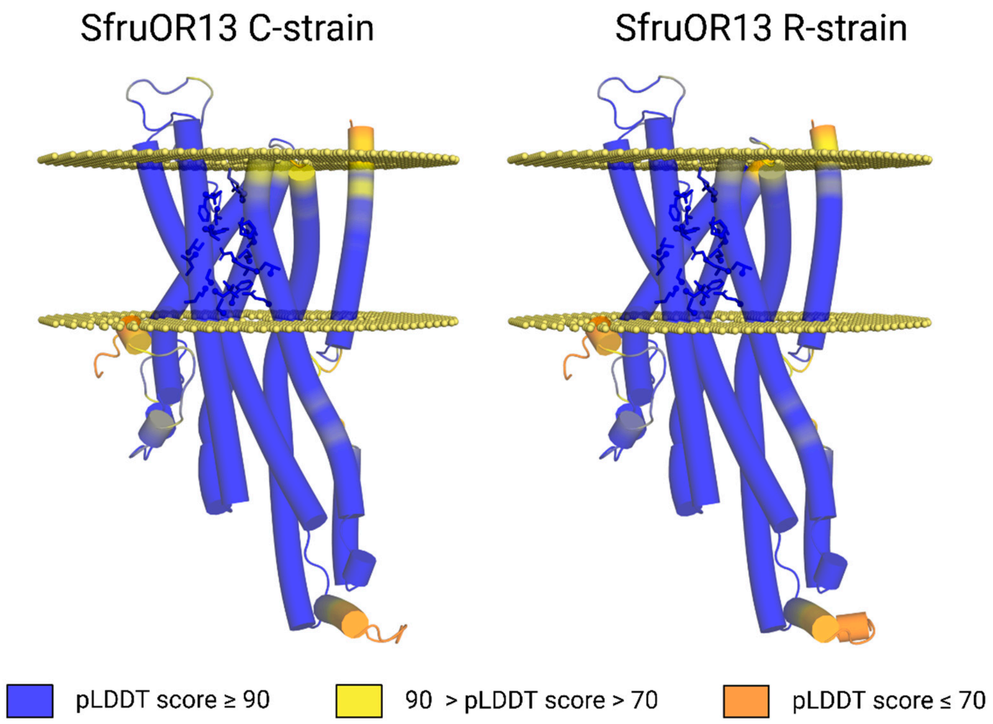

B

	SfruOR13 C-strain	SfruOR13 R-strain	AaegOR10	AgamOR28	ApisOR5	MhraOR5
**SfruOR13 C-strain**						
**SfruOR13 R-strain**	0.485 Å					
**AaegOR10**	3.574 Å	3.595 Å				
**AgamOR28**	4.134 Å	4.150 Å	2.779 Å			
**ApisOR5**	4.709 Å	4.683 Å	3.566 Å	4.063 Å		
**MhraOR5**	3.858 Å	3.787 Å	3.062 Å	3.499 Å	3.542 Å	

Figure S1. The AlphaFold2 models of SfruORs suggest high structural quality, particularly in the binding region, as highlighted by their pLDDT scores: (**A**) AlphaFold2 best models of C-SfruOR13 and R-SfruOR13 colored according to pLDDT scores: blue (≥90), yellow (70–90), and orange (<70). The side chains of the residues in the predicted binding region are shown as sticks. The receptor orientation in the membrane was determined using PPM 3.0 server [31]. This figure was generated using the molecular visualization software PyMol 2.5.4. [33] (**B**) Structural alignment of the best models of C-SfruOR13 and R-SfruOR13 with the published Cryo-EM structures of apo insect ORs [6,7,34] was performed using PyMOL. RMSD values were calculated across all receptor sequences to assess structural similarities.
